# Age-Related Differences in the Effect of Prolonged Vibration on Maximal and Rapid Force Production and Balance Ability

**DOI:** 10.3389/fphys.2020.598996

**Published:** 2020-10-29

**Authors:** Ryoichi Ema, Akihiro Kanda, Mikio Shoji, Natsuki Iida, Ryota Akagi

**Affiliations:** ^1^School of Management, Shizuoka Sangyo University, Iwata, Japan; ^2^Graduate School of Engineering and Science, Shibaura Institute of Technology, Saitama, Japan; ^3^Mizuno Corporation, Osaka, Japan; ^4^College of Systems Engineering and Science, Shibaura Institute of Technology, Saitama, Japan

**Keywords:** electromyography, plantar flexion, rate of torque development, triceps surae muscle, single-leg standing, maximal voluntary contraction, voluntary activation, V-wave

## Abstract

We tested the hypothesis that older adults would not likely experience deficits in maximal and explosive plantar flexion strength and standing balance performance induced by prolonged Achilles tendon vibration compared with young adults. Fifteen older men (OM, 73 ± 5 years) and 15 young men (YM, 24 ± 4 years) participated in two interventions on different days: lying in a quiet supine position for 30 min with or without prolonged vibration to the Achilles tendon. Before and after the interventions, maximal voluntary contraction (MVC) torque during plantar flexion, rate of torque development (RTD), and center of pressure (COP) speed during single-leg standing were measured. The root mean square of the electromyogram (RMS-EMG) during performance and V-wave and voluntary activation during MVC were assessed. The MVC torque (7 ± 7%) and RTD (16 ± 15%) of YM but not OM significantly decreased after vibration. In addition, the relative changes observed in YM positively correlated with changes in RMS-EMG of the medial gastrocnemius (MG) (MVC torque and RTD) and in MG V-wave and voluntary activation (MVC torque). COP speed significantly increased (16 ± 20%) in YM only after vibration and was accompanied by increased activation of the lateral gastrocnemius. This is the first study to show that the effects of prolonged Achilles tendon vibration on strength and balance performances were apparent in young adults only. The differences between the age groups may be related to the attenuated gastrocnemius neuromuscular function in older adults.

## Introduction

In older adults, prevention of falls is critical because a fall can cause functional disability. Age-related declines in muscular strength and balance ability are the major risk factors for falls (Rubenstein, 2006). Decreased maximal (Cattagni et al., 2014) and explosive (LaRoche et al., 2010) plantar flexor strength and a deficit in standing balance performance (Cattagni et al., 2014) may increase the risk of falls in older individuals. Large attenuations associated with aging have been reported for the above variables (Dalton et al., 2010; Thompson et al., 2014; Kurz et al., 2018). However, the neuromuscular factors underlying such attenuations are not fully elucidated. Previously, maximal (Melzer et al., 2009) and explosive (Ema et al., 2016) plantar flexor strength were correlated with the standing balance performance in older adults. It is possible, therefore, that some common neuromuscular factors related to age-related impairment of plantar flexion strength and standing balance performance exist.

The triceps surae largely contributes to the generation of plantar flexion force and the stability of body position. This muscle group comprises three muscles with different muscle fiber type compositions; the medial and lateral gastrocnemius (MG and LG), each with approximately 50% fast-twitch fibers, and the soleus (SOL) with <15% fast-twitch fibers (Johnson et al., 1973). The neuromuscular system gets impaired with aging (Gerstner et al., 2017); however, whether age-related changes in the function of the three muscles have similar or different effects on the changes in plantar flexion strength and balance performance remains unclear. Because training-induced neuromuscular adaptations were not consistent among the constituents of the triceps surae (Ema et al., 2017a), clarification of this point would provide beneficial information for the choice of better training regimens in older adults.

Age-related atrophy is characterized by preferential atrophy of fast-twitch rather than slow-twitch fibers (Lexell et al., 1988), and muscle atrophy is accompanied by a decrease in the number of motor units (Power et al., 2013). In contrast, reduced motor units by aging were not reported in the SOL (Dalton et al., 2008). Thus, reduced neuromuscular functions of the gastrocnemius, rather than the SOL, are a potential factor associated with the age-related attenuation of both plantar flexion strength and balance performance. If so, an intervention affecting the gastrocnemius neuromuscular function would change plantar flexor strength and balance performance differently between young and older adults. A previous study (Ushiyama et al., 2005) has shown decreases in maximal plantar flexion strength in young men after prolonged Achilles tendon vibration for 30 min, which was accompanied by the attenuation of the gastrocnemius but not the SOL activation during contractions. Therefore, we aimed to examine the effect of prolonged Achilles tendon vibration on maximal and rapid force production and balance ability through testing the hypothesis that compared with young adults, older adults would not likely experience vibration-induced deficits in strength and balance performance.

## Materials and Methods

### Participants and Procedures

This study was conducted after obtaining approval from the Ethics Committee of the Shibaura Institute of Technology and conducted according to the Declaration of Helsinki. A sample size estimation was performed (G^×^Power 3.1.7; Kiel University, Germany) for an expected effect size (medium, *f*^2^ = 0.25) for age-related differences in changes of MVC torque by vibration with α of 0.05, power of 0.90, and correlation among repeated measures of 0.8. The results showed that total sample was 24 (*N* = 12 in each age). In the current study, fifteen young men (YM) and 15 older men (OM) were included. Written informed consent was obtained from all participants. An independent *t*-test revealed that age and height were significantly different (*P* ≤ 0.011) between OM and YM (Table 1). None of the participants had experienced falls within the prior 6 months. All measurements were conducted with the right leg, and the ankle joint angle was maintained in the anatomical position during strength testing. The participants visited our laboratory on three different days within a week. On the first day, the participants familiarized themselves with strength and balance performance measurements. On the second and third days, the participants joined the two interventions while in the supine position for 30 min in random order: tonic vibration to the Achilles tendon (vibration condition) or no vibration (control condition), with 1–3 days between interventions. Before and after the intervention, the measurements of maximal and explosive plantar flexion strength, evoked triceps surae responses, force-plate data during single-leg standing, and surface electromyographic (EMG) amplitude during performances were obtained. A previous study (Thompson and Bélanger, 2002) has shown that the attenuation of Ia afferents activity was more than 30 min after prolonged vibration. In the present study, all measurements were completed within 11 min after the intervention.

**TABLE 1:** Physical characteristics, physical activity, and muscle thickness of the triceps surae of participants.

		**Old men (*n* = 15)**	**Young men (*n* = 15)**
Age	years	73 ± 5	24 ± 4*
Height	cm	166.5 ± 6.1	172.1 ± 4.8*
Body mass	kg	67.5 ± 9.2	67.7 ± 10.6
Physical activity			
light	min⋅day^–1^	636 ± 101	637 ± 67
moderate	min⋅day^–1^	77 ± 13	78 ± 10
vigorous	min⋅day^–1^	1 ± 1	5 ± 10
MG thickness	mm⋅kg^–1/3^	4.7 ± 0.5	5.6 ± 0.6*
LG thickness	mm⋅kg^–1/3^	3.3 ± 0.7	3.5 ± 0.5
SOL thickness	mm⋅kg^–1/3^	3.3 ± 0.8	3.5 ± 0.5

*Indicates the significant difference between older and young men. LG, lateral gastrocnemius; MG, medial gastrocnemius; SOL, soleus. Data are shown as mean ± standard deviation.

### Data Recording

During strength testing, the participants lay supine on a bench of a dynamometer (CON-TREX MJ, PHYSIOMED, Germany), and the participant’s body and foot were secured to the dynamometer with a non-elastic strap. The centers of rotation of the ankle joint and dynamometer were carefully aligned. Torque, EMG, and force-plate data were obtained at 2000 Hz using an A/D converter (PowereLab16/35, ADInstruments, Australia). All offline analyses were performed with LabChart software (ADInstruments, Australia), with being low-pass filtered at 500 Hz for torque data and band-pass filtered at 6–500 Hz for EMG data (Ema et al., 2018a). The force-plate data were low-pass filtered at 10 Hz and down sampled to 100 Hz for analyses.

### Vibration

The participants were laid supine on a bench with the dynamometer and were asked to relax during the interventions. Vibration was performed using a vibration generator (WaveMaker05, Asahi Seisakusho, Japan), with tonic vibration being applied perpendicular to the right Achilles tendon 1 cm distal from the myotendinous junction of the SOL for 30 min (Ushiyama et al., 2005). The precise location of the vibration was determined with an ultrasonic apparatus (ACUSON S2000, Siemens Medical Solutions, United States). To selectively stimulate Ia afferents, the vibration frequency was set at 80 Hz (Roll et al., 1989). The vibration force was determined using a load cell (LUR-A50NSA1, Kyowa, Japan) attached to the vibration generator. The force during vibration and peak-to-peak amplitude of the vibration were controlled at 10–15 N and 1.6 mm, respectively (Ema et al., 2017b). The current setup was in accordance with that of a previous study that reported significant decreases in the Ia afferent activity of the plantar flexors, maximal plantar flexion strength, and agonist muscle activity after intervention (Ushiyama et al., 2005).

### EMG

The surface EMG signals (Bagnoli 8 EMG System, DELSYS, United States) of the MG, LG, SOL, and tibialis anterior (TA) were recorded using pre-amplified bipolar surface electrodes (DE-2.1, 1 mm × 10 mm, 10-mm inter-electrode distance). Using real-time B-mode ultrasonography (ACUSON S2000, Siemens Medical Solutions, United States), the muscle belly and fascicle longitudinal directions were confirmed. The electrodes were placed on the muscle belly after the skin was shaved, rubbed with sandpaper and cleaned with alcohol. To match the electrode placement throughout the testing, the participants were requested to keep some marks on their skin by tracing after having a bath. The reference electrode was placed over the left lateral malleolus.

### Stimulation

According to a stimulation set up of a previous study (Ema et al., 2018b), the peak-to-peak amplitudes of the compound motor action potential (M_*max*_) of each of the triceps surae were obtained via tibial nerve stimulation in the popliteal fossa with a cathode (2 cm × 2 cm). The anode (4 cm × 5 cm) was placed over the ventral aspect of the thigh. Rectangular pulses with a duration of 0.2 ms were delivered using a constant-current variable voltage stimulator (DS7AH, Digitimer Ltd., United Kingdom). The supramaximal stimulus intensity was determined by increasing the current intensity until plateaus were achieved for the twitch torque and M_*max*_. Before and after the intervention, two M_*max*_ at a higher current (≥20%) were obtained every 10 s, and M_*max*_ was determined and averaged across the two twitches.

### Maximal Strength, Voluntary Activation (VA%), and V-Wave

The peak isometric torque was assessed during the maximal voluntary contraction (MVC) of the plantar flexors. The participants performed a warm-up procedure involving submaximal contractions before intervention. The participant exerted plantar flexion torque as forcefully as possible for 4 s twice at each time point. Two dorsiflexor MVCs before intervention were conducted to obtain the maximal TA activity as an agonist. The peak torque was normalized to the body mass. The root mean square values of the EMG signals (RMS-EMGs) of the triceps surae during MVC were determined over a 0.5-s period around the maximal torque, and were normalized to M_*max*_. The RMS-EMG of the TA during MVC was normalized to that during dorsiflexor MVC. Three seconds after the onset of contraction and 2 s after MVC, supramaximal twitch stimulation was delivered, and VA% was determined: VA% = (1 − [superimposed twitch torque/potentiated resting twitch torque]) × 100. The superimposed twitch was also used to assess V-wave. The V-wave consists of a volley of H-reflex impulses that can reach the muscle after the removal of antidromic action potential impulses by collision with descending neural drive (Duclay and Martin, 2005). Simultaneous reduction and relations with torque loss of RMS-EMG normalized to M_*max*_, VA%, and V-wave could be good evidence for a central drive limitation (Trajano et al., 2013). Thus, the peak-to-peak amplitude of the V-waves of the MG and SOL were obtained, and normalized to the equivalent measurements of the M-wave during MVC (Duclay and Martin, 2005). We did not analyze the LG wave because of the difficulty in detecting it from most participants. Although several measurements are ideal for a sufficient reliability of V-wave determination, two stimulations were performed to minimize the effects of fatigue (Trajano et al., 2013). The intraclass correlation coefficients type 1.2 of the two waves at each time point were 0.640–0.808. The means of the two contractions were used for future analyses.

### Explosive Strength

The participants conducted 10 (before the intervention) and six (after the intervention) explosive voluntary plantar flexions. The small number of contractions after the intervention was recorded to exclude the reduction of vibration effect. The participants were encouraged to exert plantar flexion force as fast and forcefully as possible for approximately 1 s, with an emphasis on fast, every 20 s. Contractions with an unstable baseline (countermovement or pre-tension) greater than 0.5 Nm in 200 ms prior to the onset of contraction were excluded from the analyses. The three contractions with the largest peak slope and peak torque >70% of the MVC torque were used for future analyses. The rate of torque development (RTD) was defined as the slope of the time-torque curve at the time points between the onset of plantar flexion and 100 ms (Ema et al., 2016) and normalized to the body mass. The RMS-EMGs of the triceps surae were calculated over the same time periods of RTD from the onset of EMG amplitude, and normalized to the M_*max*_ of each muscle. We did not obtain the RMS-EMG of TA during explosive plantar flexions because of the low repeatability of measurements for antagonist muscle activation during the explosive contractions (Ema et al., 2018a). Torque and EMG onsets were manually identified according to previously described procedures (Ema et al., 2018a). Briefly, the torque and EMG signals were viewed at a high resolution, and onset was detected as the last peak or trough within the baseline noise envelope.

### Balance Performance

Static balance performance while standing on a single-leg standing was evaluated according to a previous study (Ema et al., 2016). The participants stood barefoot with the right leg on the platform of a 40 cm × 40 cm force plate (Sports Sensing, Japan) for 30 s with their eyes open, for three trials in each testing session with 30-s rest between trials. While the participants were standing, they fixed their gaze on a visual mark located 2 m in front of them at eye level. Their arms were kept at their sides, and the left leg was knee flexed at approximately 90°. If the participants failed to keep the raised foot up or the right leg left on the platform, the trial was ended. To exclude the effect of postural changes at the beginning of the standing trials, we analyzed variables for the time corresponding to 5–30 s (Ema et al., 2016). The total center of pressure (COP) displacement during the trial was determined and divided by 25 s (COP speed) and normalized to height. We investigated COP speed because COP speed was sensitive to detect age-related differences (Qiu and Xiong, 2015) and a good day-to-day repeatability was confirmed in our previous study (Ema et al., 2017a). The lowest COP speed obtained in each testing session while the participant was standing for 30 s was used for later analyses. If the participant could not stand for 30 s, the trial in which the participant could stand the longest was used for the analysis, and the variables were determined 5 s after the onset until 3 s before the end of the trial (Ema et al., 2016). We determined the RMS-EMGs of the investigated muscles corresponding to the time periods of COP displacement and normalized to those during MVC.

### Muscle Thickness

Using B-mode ultrasonography, the thicknesses of the three muscles were determined during quiet standing at 30% of the lower leg length from the popliteal crease to the lateral malleolus and 50% of the mediolateral width for the MG and LG. The thickness of the SOL was determined using the same images of the LG. Muscle thickness was defined as the mean of the distances between superficial and deep aponeuroses measured at both ends of each image with a width of 45 mm, and normalized to one-third power of the body mass. The measurement was performed three times. The analyses were conducted using Image J (National Institutes of Health, United States) and the mean value was used for future analyses.

### Habitual Physical Activity

After the completion of the testing sessions, the participants were requested to conduct their routine daily activities while wearing a device (Active style Pro HJA-750C, Omron Health Care, Japan) for 10 days. We instructed the participants to wear the device while performing activities of daily living, except for the time spent in bathing or sleeping. If the participants did not wear the device for at least 500 min a day, the data were excluded from the analyses. The mean of days for analyses was 9 days for both OM and YM (range: from 4 to 10 days), which was sufficient to meet the criteria for further analyses (Watanabe et al., 2020). The mean magnitude of physical activity was determined at three levels: low [<3.0 metabolic equivalents (METs)], moderate (3.0–5.9 METs), and vigorous intensities (>6.0 METs) (Ema et al., 2017a).

### Statistical Analyses

Statistical analyses were performed using the SPSS version 25 (IBM, United States). The normality of data distribution was examined using the Shapiro–Wilk test. When the normality assumption was violated, the data were log-transformed. All data are shown as means ± SD of raw data to facilitate interpretation. A three-way analysis of variance (ANOVA) with one between-group factor [age (older, young)] and two within-group factors [time (before and after) and intervention (vibration and control)] was conducted for dependent variables except for muscle thickness. A two-way ANOVA [age × muscle (MG, LG, SOL) or muscle (MG, LG, SOL, TA), age × intensity (light, moderate, vigorous)] was performed with muscle thickness, RMS-EMG during single-leg standing at baseline (average of the control and vibration conditions) and physical activity as dependent variables. When a significant interaction was detected, follow-up ANOVAs with Bonferroni multiple-comparisons were performed. If significant changes in the strength and/or COP speed were observed, Pearson product moment correlation was used to examine the relationships between the changes in variables. *P* < 0.05 was considered statistically significant. For an effect size (ES) index, Cohen’s d in between-subject designs (Lakens, 2013) was calculated using the between-age differences in changes by intervention between vibration and control conditions. We obtained 90% confidence intervals (Hopkins, 2007). The thresholds were 0.20, 0.60, and 1.20 for small, moderate, and large, respectively (Hopkins et al., 2009). When the ES was ≥0.20 and the lower limit of the 90% confidence intervals for ES was ≥−0.20 with a significant change in at least one of the age-groups, the vibration effect was considered substantially different between the ages.

## Results

We excluded two OM data from the analysis of variables during single-leg standing because the participants could not stand for 10 s straight in at least one testing session. As for the V-wave of the MG and SOL, we could not detect the wave in five and two OM, respectively. Because of some mechanical accidents, data of EMG amplitude of LG during single-leg standing for one YM and physical activity for two YM were not obtained; therefore, the number of participants was decreased for the variables.

### Variables During MVC

**FIGURE 1:**
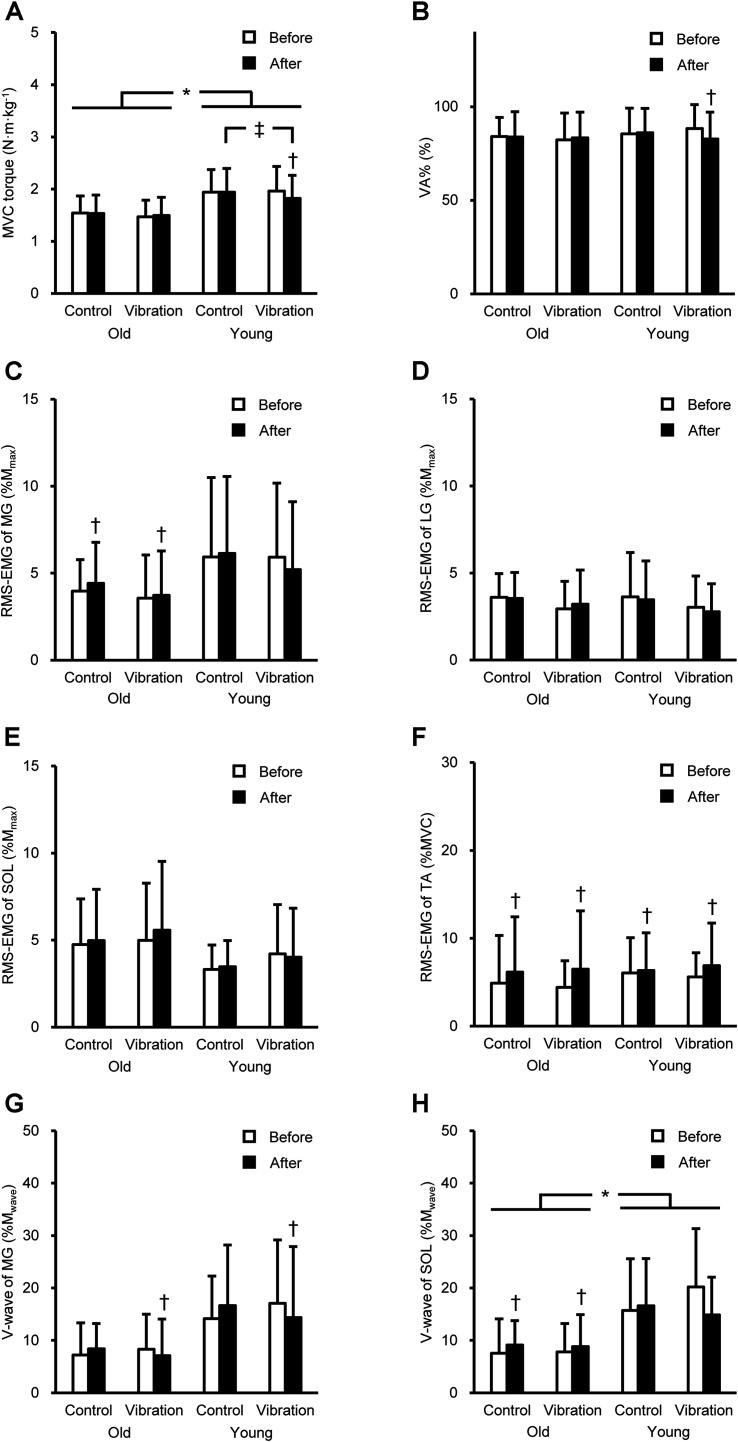
Peak torque during maximal voluntary contraction (MVC, **A**) of plantar flexion, voluntary activation (VA%, **B**), root mean square of the electromyogram (RMS-EMG) of the medial gastrocnemius (MG, **C**), lateral gastrocnemius (LG, **D**), soleus (SOL, **E**), and tibialis anterior (TA, **F**), and V-wave of MG **(G)** and SOL **(H)** during plantar flexor MVC before and after the intervention. Data are shown as mean + standard deviation. RMS-EMGs of the triceps surae and V-waves were normalized to the peak-to-peak compound muscle action potential amplitude at rest (M_*max*_) and during MVC (M_*wave*_), and RMS-EMG of TA was normalized to that during dorsiflexor MVC. *Indicates a significant difference between older and young men. † Denotes a significant change after the intervention. ‡Shows a significant difference between control and vibration conditions.

Figure 1 shows the peak torque and neuromuscular variables during plantar flexor MVC. A significant age × intervention × time interaction was found for peak torque [*F*(1,28) = 7.955, *P* = 0.009, partial η^2^ = 0.221] and VA% [*F*(1,28) = 4.344, *P* = 0.046, partial η^2^ = 0.134]. On average, the torque value was 23% higher in YM than in OM (*P* = 0.009–0.032). For YM, the torque significantly (*P* = 0.001) decreased after the vibration by 7 ± 7%, and the torque after the vibration was significantly (*P* = 0.011) lower than that with no vibration. The VA% significantly (*P* = 0.006) decreased after tendon vibration by 6 ± 7% in YM. A significant age × time interaction [*F*(1,28) = 6.705, *P* = 0.015, partial η^2^ = 0.193] for MG RMS-EMG and main effect of time [*F*(1,28) = 18.086, *P* < 0.001, partial η^2^ = 0.392] for TA RMS-EMG were observed. The RMS-EMGs were significantly higher after than before in both conditions for MG of OM (*P* = 0.045) and for TA of both ages. For V-wave, there were significant interactions of time × intervention [*F*(1,23) = 8.164, *P* = 0.009, partial η^2^ = 0.262] for MG and age × time [*F*(1,26) = 11.999, *P* = 0.002, partial η^2^ = 0.316] for SOL. The MG V-wave significantly (*P* = 0.026) decreased after the tendon vibration in both ages. The SOL V-wave was significantly (*P* = 0.002) higher after than before in both conditions for OM, and was significantly (*P* ≤ 0.004) higher in YM than in OM. The dorsiflexor MVC torque was significantly (*P* = 0.001) higher in YM (vibration, 0.57 ± 0.07 N⋅m⋅kg^–1^; control, 0.56 ± 0.07 N⋅m⋅kg^–1^) than in OM (vibration, 0.47 ± 0.08 N⋅m⋅kg^–1^; control, 0.46 ± 0.09 N⋅m⋅kg^–1^).

### Variables During Explosive Contractions

**FIGURE 2:**
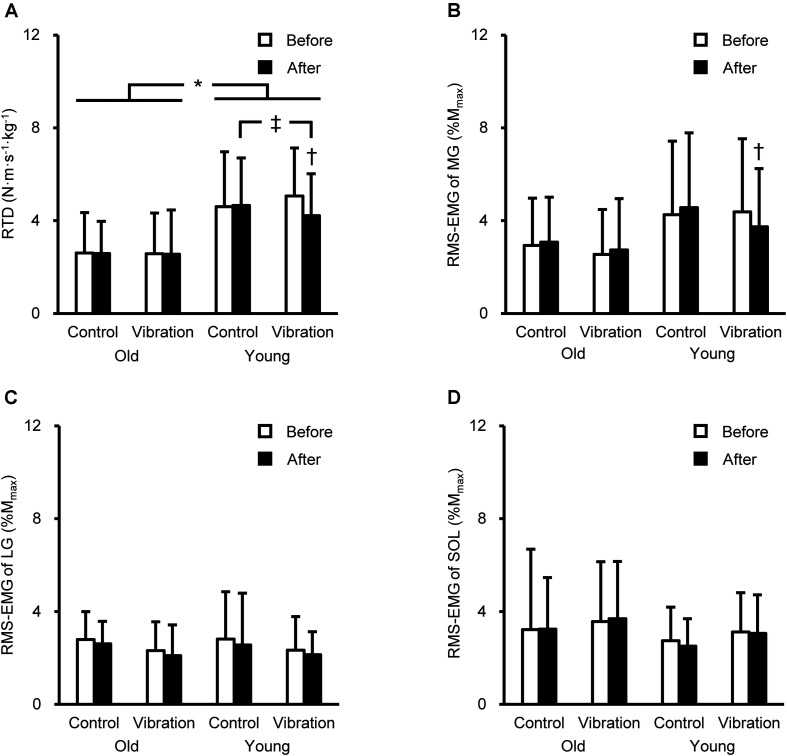
Rate of torque development (RTD, **A**) of plantar flexion and root mean square of the electromyogram (RMS-EMG) during explosive contraction of the medial gastrocnemius (MG, **B**), lateral gastrocnemius (LG, **C**), and soleus (SOL, **D**) before and after the intervention. Data are shown as mean + standard deviation. RTD was defined as the slope of the time-torque curve during time intervals of 0–100 ms from the onset of plantar flexion. RMS-EMGs of the triceps surae were normalized to the peak-to-peak compound muscle action potential amplitude at rest (M_*max*_). *Indicates a significant difference between older and young men. † Denotes a significant change after the intervention. ‡Shows a significant difference between control and vibration conditions.

The RTD and RMS-EMGs during explosive contractions are presented in Figure 2. A significant age × intervention × time interaction for RTD [*F*(1,28) = 4.265, *P* = 0.048, partial η^2^ = 0.132] and MG RMS-EMG during 0–100 ms [*F*(1,28) = 4.561, *P* = 0.042, partial η^2^ = 0.140] was observed. On average, the RTD was 46% higher in YM than in OM (*P* = 0.003–0.021). YM showed lower RTD after than before the vibration (16 ± 15%) and after the quiet supine (*P* ≤ 0.045). The MG RMS-EMG during 0–100 ms significantly (*P* = 0.009) decreased by 10 ± 21% in vibration condition for YM, but not OM.

### Variables During Single-Leg Standing

**FIGURE 3:**
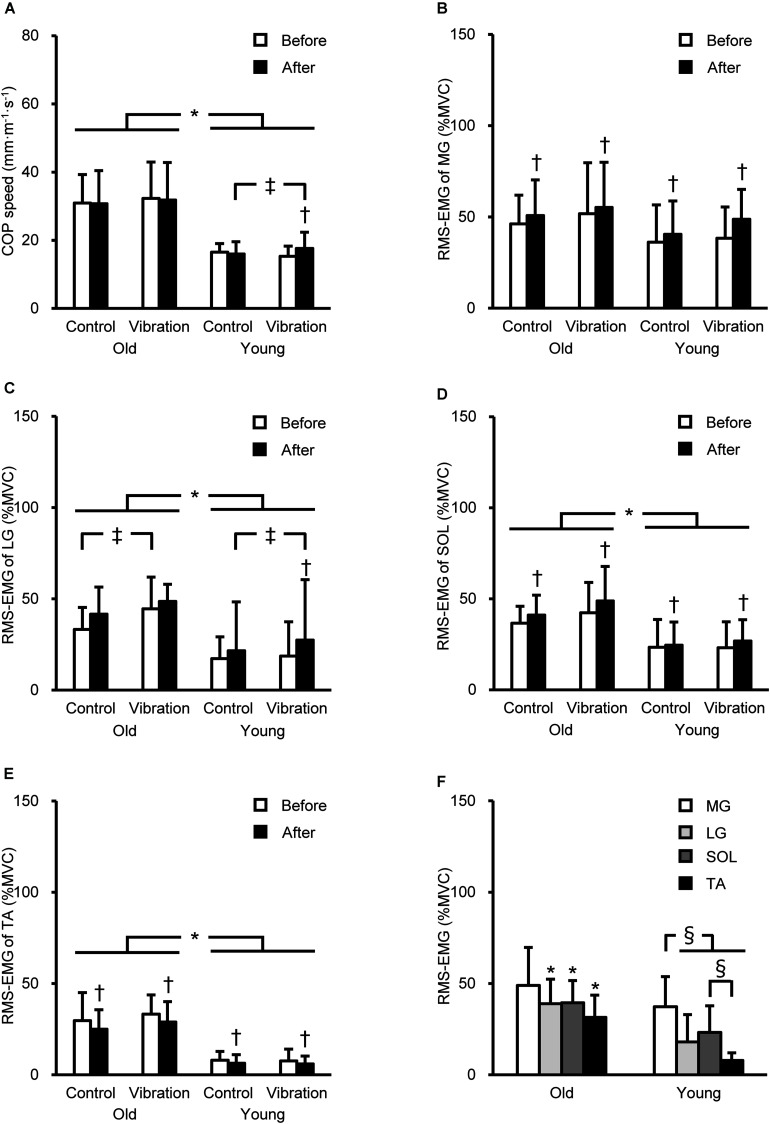
Center of pressure (COP) speed **(A)** and root mean square of the electromyogram (RMS-EMG) of the medial gastrocnemius (MG, **B**), lateral gastrocnemius (LG, **C**), soleus (SOL, **D**), and tibialis anterior (TA, **E**) during single-leg standing before and after the intervention, and RMS-EMGs before the intervention (means of the control and vibration conditions, **F**). RMS-EMGs were normalized to those during maximal voluntary contraction (MVC) as agonist (%MVC). *Indicates a significant difference between older and young men. † Denotes a significant change after the intervention. ‡Shows a significant difference between control and vibration conditions. §Demonstrates a significant difference between muscles.

Three-way ANOVA showed a significant (*P* ≤ 0.041) age × intervention × time interaction for COP speed [*F*(1,26) = 5.427, *P* = 0.028, partial η^2^ = 0.173] and LG RMS-EMG [*F*(1,25) = 4.627, *P* = 0.041, partial η^2^ = 0.156], the main effect of time for MG RMS-EMG [*F*(1,26) = 13.109, *P* = 0.001, partial η^2^ = 0.335], and age and time for RMS-EMGs of SOL [age, *F*(1,26) = 13.950, *P* = 0.001, partial η^2^ = 0.349; time, *F*(1,26) = 5.222, *P* = 0.031, partial η^2^ = 0.167] and TA [age, *F*(1,26) = 72.372, *P* < 0.001, partial η^2^ = 0.736; time, *F*(1,26) = 7.552, *P* = 0.011, partial η^2^ = 0.225]. The COP speed and RMS-EMGs of LG, SOL, and TA were significantly (*P* ≤ 0.002) higher in OM than in YM (Figure 3). The COP speed significantly (*P* = 0.010) increased after the tendon vibration by 16 ± 20% only in YM. Regarding RMS-EMGs before the intervention, a significant [*F*(3,75) = 7.006, *P* < 0.001, partial η^2^ = 0.219] age × muscle interaction was observed. Except for MG RMS-EMG, these were significantly (*P* ≤ 0.002) higher in OM than in YM, and in YM, MG RMS-EMG was highest among the four muscles (*P* ≤ 0.007).

### Relationships Between Vibration-Induced Changes in Variables

**FIGURE 4:**
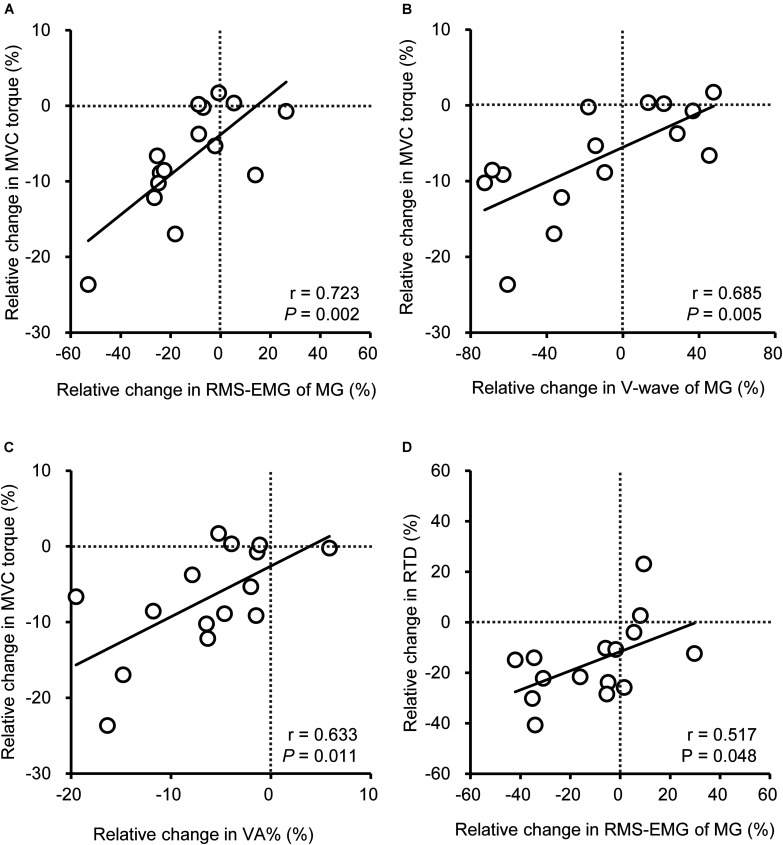
Relationship among vibration-induced relative changes in peak torque during maximal voluntary contraction (MVC, **A–C**) of plantar flexion or rate of torque development from the onset to 0–100 ms (RTD, **D**) and neural variables [root mean square of the electromyogram (RMS-EMG), voluntary activation (VA%), V-wave] of the medial gastrocnemius (MG) for young men. Data of older men were not included because no significant changes were shown for MVC and RTD after the vibration.

As shown previously, a significant change in the strength and COP speed after vibration was observed in YM only. Therefore, Pearson product moment correlation was used to indicate the relationships between the vibration-induced relative changes in variables for YM. The change in MVC torque was positively correlated (*r* = 0.633–0.723; *P* = 0.002–0.011) with the changes in MG RMS-EMG, MG V-wave, and VA% (Figure 4), and a positive correlation was observed between changes in VA% and MG RMS-EMG (*r* = 0.640; *P* = 0.010). There was a positive correlation between the changes in MG RMS-EMG during 0–100 ms and RTD (*r* = 0.517; *P* = 0.048). There were no significant relationships between the changes in COP speed and RMS-EMGs.

### Muscle Thickness

A significant [*F*(2,56) = 3.339, *P* = 0.043, partial η^2^ = 0.107] age × muscle interaction showed that the MG thickness was significantly (*P* = 0.001) smaller by 15% in OM than in YM (Table 1). In contrast, no significant age-related differences were found for LG or SOL thickness.

### Habitual Physical Activity

The magnitudes of physical activity are shown in (Table 1). Age did not have any significant effect and an interaction of age × muscle was not found.

### Interpretation of the Difference in the Prolonged Vibration Effect Between Ages

Regarding the variables that changed significantly in each age group, the ES and its uncertainty are shown in Table 2. Small-to-moderate effects were observed among the variables, whereas some were not substantial. We refer to the interpretations for some variables. For RMS-EMG of MG during MVC, the value for OM was significantly increased and no significant change was shown for YM after the intervention, but the ES reached a substantial level. An interaction was not significant for RMS-EMGs of MG and LG during standing, whereas the ESs were small-to-moderate. These show that the vibration effect on the variables was substantially different between the age groups.

**TABLE 2:** Difference in the effect of prolonged vibration between older and young men.

**Cohen’s d**		**90% confidence interval**
**Maximal strength variables**		
MVC torque	1.03*	0.41 to 1.70
RMS-EMG of MG	0.42*	−0.20 to 1.00
RMS-EMG of TA	0.06	−0.56 to 0.68
VA%	0.76*	0.14 to 1.40
V-wave of MG	0.33	−0.37 to 1.00
V-wave of SOL	0.44	−0.21 to 1.10
**Explosive strength variables**		
RTD	0.75*	0.13 to 1.40
RMS-EMG of MG	0.78*	0.16 to 1.40
**Balance performance variables**		
COP speed	0.62*	−0.03 to 1.30
RMS-EMG of MG	0.59*	−0.07 to 1.30
RMS-EMG of LG	0.69*	0.03 to 1.30
RMS-EMG of SOL	0.06	−0.58 to 0.71
RMS-EMG of TA	0.06	−0.58 to 0.71

*Indicates that the effect of prolonged vibration was substantially different between older and young men. COP, center of pressure; LG, lateral gastrocnemius; MG, medial gastrocnemius; MVC, maximal voluntary contraction; RMS-EMG, root mean square; RTD, rate of torque development; SOL, soleus; TA, tibialis anterior; VA%, voluntary activation.

## Discussion

The present study showed that prolonged Achilles tendon vibration decreased the plantar flexor MVC torque and RTD and increased COP speed in YM, whereas OM did not show any vibration-induced changes in the variables. These results support our hypothesis. The current study revealed that the effects of prolonged vibration on maximal and explosive strength and balance performance substantially differ between older and young adults.

Prolonged vibration-induced changes of the MVC torque and RTD in YM were positively correlated with the change of MG neural variables (Figure 4), suggesting that the decrease in torque value is attributed to impairment in MG neuromuscular activation. It is noted that reduction in the MVC torque was unlikely to have occurred due to changes in antagonist TA activation considering that the intervention did not have any effect on the activation. Although the V-wave of MG was reduced by vibration for OM and YM, insignificant changes in MVC torque and RTD for OM show that the impact of change in MG activation for OM was small. An explanation for the lesser vibration effect in OM is that the ability of MG recruitment during contractions was already attenuated by aging at baseline; therefore, prolonged vibration would have little effect on strength and MG activity. Bongiovanni et al. (1990) observed that discharge rate during MVC was reduced by prolonged vibration for high-threshold rather than low-threshold motor units. Consistent with the current study, older adults displayed no changes in knee extensor MVC torque and quadriceps femoris EMGs following a prolonged infrapatellar tendon vibration (Richardson et al., 2006), who proposed that a gamma-loop function, which plays an important role to recruit type II fibers, was impaired for older adults, and thus vibration produced less effect. The smaller MG size in OM (Table 1) may be associated with the age-induced loss of motor units (Power et al., 2013), particularly those relate to the recruitment of type II fibers, whereas the number of SOL motor units was not affected by aging (Dalton et al., 2008). Therefore, an age-related impairment of the ability to recruit high-threshold motor units in OM may be related to insignificant effects of vibration, whereas YM may have experienced the difficulty in recruitment of high-threshold motor units of MG by prolonged vibration.

For YM, the relative change in MVC torque was positively correlated with changes not only in MG RMS-EMG but also in MG V-wave and VA% (Figure 4), and the change in MG RMS-EMG but not in the other two muscles, was positively correlated with the change in VA%. These consistent changes in the three neural variables (RMS-EMG/M_*max*_, V-wave, and VA%) suggested a reduced central drive of MG affecting force production, rather than peripheral factors (Trajano et al., 2013). However, it is difficult to specify the levels of origin of the central drive limitation by vibration. There is not a consensus on the prolonged vibration-induced changes in cortico-spinal excitability due to confounding changes in variables at both spinal and supra-spinal levels (Souron et al., 2017b). Souron et al. (2019) showed that prolonged vibration decreased the spinal excitability, being related to the decrease in intrinsic motor neuron excitability rather than presynaptic inhibition. In contrast, cortical excitability increased due to prolonged vibration to compensate for the reduced spinal excitability (Souron et al., 2017a). Such changes might have occurred for YM in the current study. Future studies are needed to clarify the cortico-spinal excitability.

The vibration effects on COP speed was substantially different between OM and YM. A previous study (Ema et al., 2017a) has suggested that a training-induced decrease in triceps surae activations during postural tasks and an improvement in RTD at early phase (from onset to ∼150 ms) beneficially affected static balance performance. In other words, opposite responses of them induced by an intervention can cause the balance performance deficit. In the current study, YM showed vibration-induced decrease of RTD and an increase of LG activation. Although the interaction was not significant, the vibration effect on RMS-EMG of MG during single-leg standing was substantially different between OM and YM (Table 2), indicating the greater increase of MG activation by vibration for YM than OM. Moreover, the magnitude of MG activation during standing was greatest in YM (Figure 3F), showing that young individuals largely rely on MG for postural control. Therefore, vibration effect on MG activation may have affected COP speed. Taken together, the substantial between-age differences in the effect of vibration on gastrocnemius activations during single-leg standing and on RTD (Table 2) may be associated with increased COP speed in YM.

Root mean square of the electromyogram of LG in OM was different between conditions at baseline. Because all participants joined a familiarization session and the order of condition was random, the substantial effect of practice is unlikely, although the reason for the difference is difficult to be ascertained. In addition, some variables changed in control condition as well as vibration condition. In both conditions, participants were in a quiet, supine position for 30 min. Thus, the effect of prolonged maintenance of posture on the variables might have been larger than those of prolonged Achilles tendon vibration. Interestingly, the increase of RMS-EMG in both conditions was also observed for antagonist TA during plantar flexor MVC. It might be possible that reciprocal Ia inhibition between agonist and antagonist muscles (Katz et al., 1991) was impaired by the posture maintenance.

A limitation of the current study is that H-reflex was not obtained; therefore, vibration-induced changes in spinal excitability were not elucidated in the current study. Previous studies have observed the vibration-induced decrement of H-reflex without the corresponding changes in strength (Fry and Folland, 2014) and/or agonist EMG (Ushiyama et al., 2005), indicating that reduced spinal excitability does not substantially affect strength performance. This may be partly explained by the compensatory increase in cortical excitability by vibration (Souron et al., 2017a). In addition, we obtained SOL EMG from one site. Because SOL comprises four compartments (Bolsterlee et al., 2018), the findings may be specific to the investigated compartment in the current study.

In summary, the present study is the first to show that the effects of Achilles tendon vibration on the strength and balance performances substantially differed between OM and YM. The vibration-induced impairments in strength and balance performance were notable in YM, whereas performance changes were not observed in OM. The attenuation observed in YM was considered to be accompanied by the neuromuscular changes in the gastrocnemius, particularly MG for strength and LG for single-leg standing. The lack of vibration-induced changes in performance in OM may be attributed to the attenuated function of the gastrocnemius.

## Data Availability Statement

The raw data supporting the conclusions of this article will be made available by the authors, without undue reservation.

## Ethics Statement

The studies involving human participants were reviewed and approved by the Ethics Committee of the Shibaura Institute of Technology. The patients/participants provided their written informed consent to participate in this study.

## Author Contributions

RE and RA conceived and designed the experiments. RE, AK, MS, and NI performed the experiment. RE, AK, and MS analyzed the data. RE drafted the manuscript and prepared tables and figures. All authors interpreted the results of the research, edited, critically revised, and approved the final version of the manuscript, and have agreed to be accountable for all aspects of the work related to its accuracy and integrity.

## Conflict of Interest

AK was employed by Mizuno Corporation. The remaining authors declare that the research was conducted in the absence of any commercial or financial relationships that could be construed as a potential conflict of interest.
